# Filamentous crystal growth in organic liquids and selection of crystal morphology

**DOI:** 10.1038/s41598-022-13851-5

**Published:** 2022-06-15

**Authors:** Takumi Yashima, Marie Tani, Rei Kurita

**Affiliations:** Department of Physics, Tokyo Metropolitan University, 1-1 Minamioosawa, Hachiouji, Tokyo 192-0397 Japan

**Keywords:** Statistical physics, thermodynamics and nonlinear dynamics, Phase transitions and critical phenomena, Chemical physics

## Abstract

Filamentous crystals such as whisker crystals are often seen not only in metallic liquids, but also in organic liquids and solutions. They are interesting as reinforce materials. However, it remains challenging to induce filamentous crystals due to an incomplete understanding of the mechanisms behind their formation. In this paper, we investigate filamentous crystal growth in viscous organic liquids. It is found that filamentous crystals grow via an extraordinary dynamical path, where the molecules locally evaporate to bubbles and then redeposite to the tip of growing crystalline filaments. We also succeeded in controlling whether filamentous or faceted crystal growth is selected by inducing or suppressing the bubbles.

Crystalline materials form the base of most of the functional electronics, making the development of new crystals a focal point for research over the years^1–4^. The electronic states and physical characteristics of crystals are not only determined by microscopic crystal structure, but also by macroscopic morphology, as seen in topological effects^3^, the alignment of carbon nanotubes^5^, and polymer crystals^4,6^. An example of this is the monocrystalline whisker: whiskers are single, defect-free filaments of materials^7–9^. Whisker crystals can be ubiquitously seen in many materials, for example, in metallic liquids such as zinc and tin^7^, in fullerenes^8^, and even in organic viscous liquids^9^. It is known that whiskers have their high tensile strength and that whisker of the fullerenes is stable to the electron beam irradiation^8^. Though whiskers are potentially useful, the growth of whiskers may also induce failure in the insulation of electronic equipment. In order to gain control over crystal morphology, it is necessary to understand the mechanism by which they form.

It seems that mechanism of filamentous crystal growth depends on the systems. In metallic system, filamentous crystal usually grows from the crystal base and it is induced by mechanical stress^7^. Meanwhile, the filamentous crystal growth of salts with impurities or with gypsum occurs by anisotropic concentration effect^10,11^. Interestingly, the whisker of the fullerene is induced at a liquid-liquid interface between two solvents^8^. In addition, the filamentous crystal growth has been observed instead of faceted crystal growth in supercooled viscous liquids such as o-terphenyl (OTP) and indomethacin in certain temperature ranges^9^. Although whisker crystals are by no means rare, the mechanism behind filamentous crystal growth is not yet fully understood.

Here, we investigate the mechanism behind filamentous crystal growth in organic liquids, OTP and salol, and propose a new way to control crystal morphology. It is found that filaments grow via an extraordinary dynamical pathway i.e. through the formation of bubbles (Fig. 1a). Furthermore, we report success in artificially controlling the morphology, switching from filaments to faceted crystals, by suppressing the bubbles.

## Results

### Emergence of bubbles during crystallization

**Figure 1:**
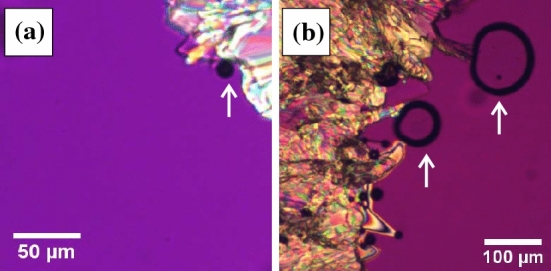
Microscopy observation of crystal growth in (**a**) OTP at 283 K and (**b**) salol at 278 K. The arrows in (**a**,**b**) indicate the emerged bubble during the crystal growth.

We start with microscopy observation of emergence of bubbles during crystallization. Figure 1 shows microscopy observation during crystallization of (a) OTP at 283 K and (b) salol at 278 K. While the crystal front grows, it is often the case that there is a bubble located between the liquid and the crystal i.e. the black droplet seen in Fig. 1a,b. It is found that the bubbles appear at grain boundaries in the interior of the crystal. In addition, when the crystal is melted, the emerged bubbles mostly disappear within several seconds.

Here, we note a possibility of the origin of the emerged bubbles, although it is hard to specify components inside the bubble in general. It is known that OTP and salol have large density differences between liquid and crystal ($0.10\,\hbox {g}/\hbox {cm}^3$ for OTP at 283 K and $0.14 \,\hbox {g}/\hbox {cm}^3$ for salol at 278 K^12^). Since the density should be conserved locally, the density of the liquid at the interface decreases, especially at the region sandwiched between the crystals. Thus, cavitation can occur due to the negative pressure^13,14^. This scenario is consistent with the fact that most bubbles disappear when the crystal melts. Meanwhile, it is known that the bubble does not emerge during crystallization in triphenyl phosphite^15^. The density differences between liquid and crystal in triphenyl phosphite is $0.05 \,\hbox {g}/\hbox {cm}^3$ at 220 K^16^. This also supports that the bubbles in OTP and salol are emerged because of the density difference.

Meanwhile, some gas components can be solved in the liquid. Since the crystal cannot include the impurities, the impurities are ejected to the outer of the crystal. Thus, the bubble may include those gas components.

### Filamentous crystal growth with the emerged bubble

**Figure 2:**
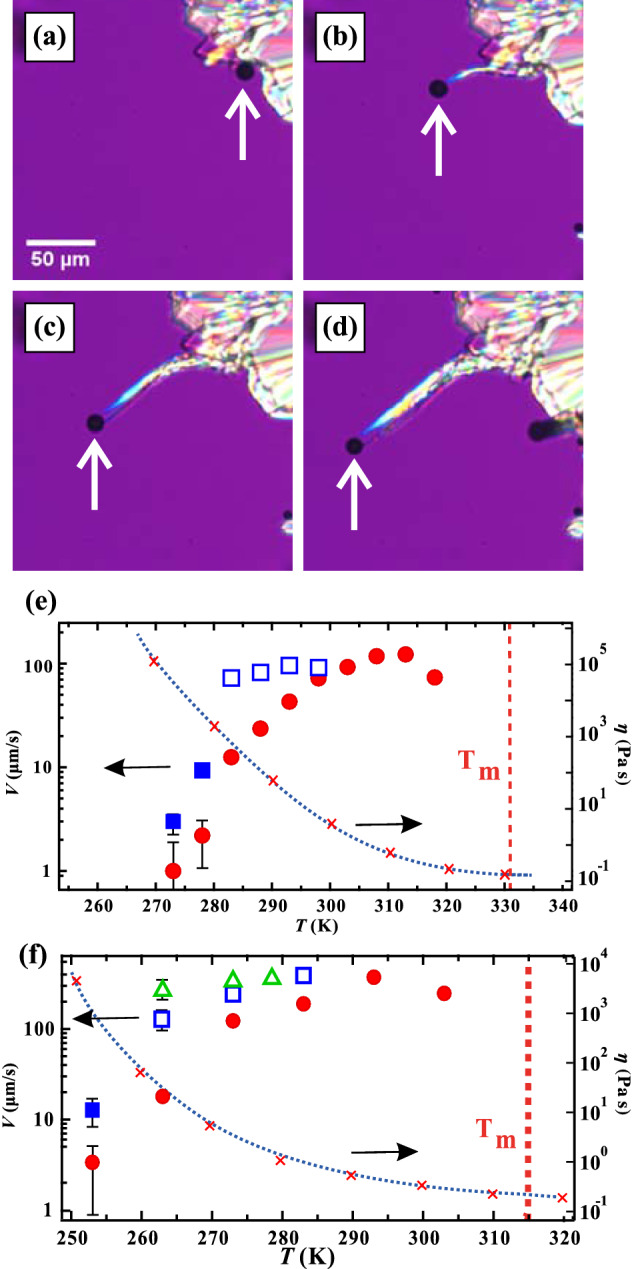
Microscopy observation of filamentous crystal growth in OTP at 283 K. (**a**) $t = 27.0 \,\hbox {s}$, (**b**) 28.0 s, (**c**) 29.0 s, and (**d**) 30.0 s. The arrow indicates the bubble which induces filamentous crystal growth. (**e**) The growth rate of the OTP crystal and (**f**) the growth rate of the salol crystal as a function of temperature. Filled circles correspond to crystal growth in bulk. Open squares in (**e**) correspond to filamentous growth with the stable bubble, while filled squares correspond to filamentous growth with the unstable bubble. Open triangles, and open squares in (**f**) correspond to the crystal growth with the large bubble (100–120 $\upmu$m), and with the small bubble (40–60 $\upmu$m), respectively. *V* is obtained by averaging over 5 points. The errors in *V* are described as the error bars, while the error bars are not described if the error is smaller than the symbol size. The growth rate of the filamentous crystal with the bubble (open squares) remains almost constant between 283 and 303 K, although *V* in the bulk (filled circles) decreases due to increase of the viscosity. It is also found that the filamentous crystal growth rate suddenly decreases when the bubbles are unstable. Cross symbols correspond to the viscosity. The viscosity data of OTP and salol digitized from the figures in Refs.^17,18^. The blue dotted line is eye guide for the viscosity.

**Figure 3:**
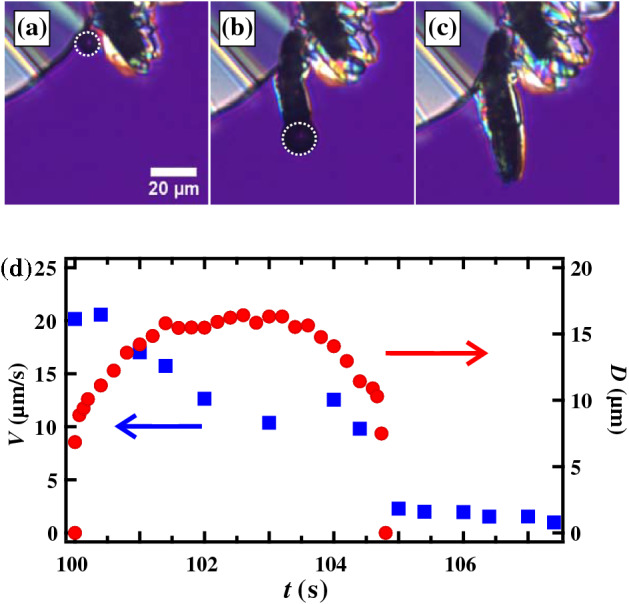
Microscopy observation of filamentous crystal growth in OTP at 278 K. (**a**) $t = 100.0 \,\hbox {s}$, (**b**) 103.0 s, and (**c**) 105.5 s. The dotted line indicates the bubble, which induces filamentous crystal growth. (**d**) The time evolution of the growth rate of the filamentous crystal (squares) and the diameter of the bubble (circles). The bubble disappears at $t = 105.5 \,\hbox {s}$; the growth rate also suddenly decays toward the bulk growth rate. The measurement errors are smaller than the symbol size.

Then we show filamentous crystal growth with the emerged bubble (Fig. 2; Movie S1). Figure 2a–d show the crystal growth of OTP at 283 K. A filamentous crystal goes on to form which follows the bubble’s movement (Fig. 2b–d). It is worth noting that the filamentous crystal growth is faster than the bulk crystal growth. We note that these bubbles are stable at the tip of the crystal over the range 283–298 K. Above 298 K, bubbles appear at grain boundaries in the interior of the crystals and are trapped inside. On the other hand, below 283 K, the number of bubbles decreases significantly and disappear in a few seconds even if they appear. Figure 3a–c show the microscopy images at 278 K. A small bubble appears with some radius at $t = 100.0 \,\hbox {s}$. This size is maintained for several seconds before the bubble disappears (Fig. 3c). Figure 3d shows the time evolution of the bubble diameter and a crystal growth rate. The crystal growth rate is faster than that in bulk, but it suddenly decreases after the bubble disappears. It also supports that the emerged bubble induces the filamentous crystal growth.

To confirm the reproducibility of the filamentous crystal growth in bulk, we checked for filamentous crystal growth in an open system where the OTP liquid is dropped on a cooled substrate. Bubbles are also generated in the open system, with filamentous crystal growth following bubble movement (see Movie S2).

**Figure 4:**
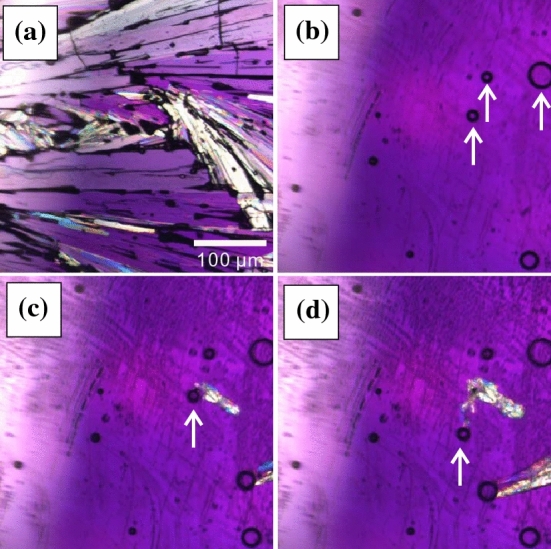
(**a**) A snapshot of a crystal. (**b**) The crystal was heated above the melting temperature and then the crystal was melted . The emerged bubbles remain indicated as arrows. Then, the liquid was quenched at 283 K. A crystal was nucleated and grew with time. (**c**) A snapshot just before the crystal was contacted with the remained bubble ($t = 7.2 \,\hbox {s}$). (**d**) A filamentous growth was observed after the crystal was contacted with the remained bubble ($t = 8.3 \,\hbox {s}$).

Here, it should be clarified whether the filamentous crystal grows and causes the bubble to appear, or vice versa. Bubbles emerged during crystallization rarely remain in the liquid phase after the crystal is melted above the melting point (see Fig. 4a,b). When this is quenched again to the crystallization temperature, a crystal is nucleated and it grows with time (Fig. 4c). The growth front makes contact with these bubbles, initiating filamentous crystal growth (Fig. 4d; Movie S3). We note that the bubble also starts moving just after contact with the growing crystal interface. In observations of such situations, we can confirm the crystal growth rate is similar to the rate of filament growth when bubbles were found already at crystal interfaces, like for the velocities denoted by the open squares in Fig. 2f. From those results, we confirmed that the emerged bubbles induce the filamentous crystal growth. We note here that those remaining bubbles can be ubiquitously observed even though a size of the bubble is nanoscale^19–22^. Mechanism of the stability of the nano bubbles in the liquid has also been unsolved yet.

### Growth rate of filamentous crystal

Then we investigate the temperature dependence of the growth rate of the filamentous crystal. The growth rate *V* is obtained by averaging over 5 different points, either on the tip of different filaments or on a growth front. Figure 2e show the temperature dependence of *V* in OTP. The red filled circles show the growth rate in the bulk region (without bubbles). Open squares correspond to filamentous growth with the stable bubble, while filled squares correspond to filamentous growth with the unstable bubble. In OTP system, the sizes of the emerged bubbles are similar (about $10 \,\upmu \hbox {m}$) and the error in the growth rate is $\pm 1 \,\upmu \hbox {m/s}$. Meanwhile, the sizes of the emerged bubbles are distributed in the salol system. Open triangles and open squares in Fig. 2f correspond to the crystal growth with the stable large bubble (100–120  $\mu \hbox {m}$), and with the stable small bubble (40–60 $\,\upmu \hbox {m}$), respectively. The error in *V* comes from the bubble size dependence. It is found that the growth of the filamentous crystal is faster when the bubble size is larger.

Here, it is remarkable that the growth rate *V* of the filaments remains almost constant between 283 and 298 K in OTP and between 263 and 283 K in salol. The viscosities in OTP and salol dramatically increase with decreasing temperature shown as in Fig. 2e,f^17,18^. This suggests that the growth of filaments with bubbles only weakly depends on the viscosity, while the temperature dependence of the growth rate in bulk is bell-shaped and seems to be consistent with classical theory^1,2^.

### Filamentous crystal growth in the capillary tube

We go on to investigate the mechanism of the filamentous crystal growth. Since the bubble is emerged from the liquid of OTP or salol, it is natural to think that the molecules are deposited onto the crystal surface from the bubble, while molecules are simultaneously transferred from the liquid to the bubble via evaporation. In this case, the position of the bubble center changes due to deposition and evaporation. This position change would be induced in an Ising-like manner rather than a diffusive manner; this is consistent with the fact that the velocity of the bubble, which is equal to the growth rate of the filamentous crystal growth, is less dependent on the viscosity.

**Figure 5:**
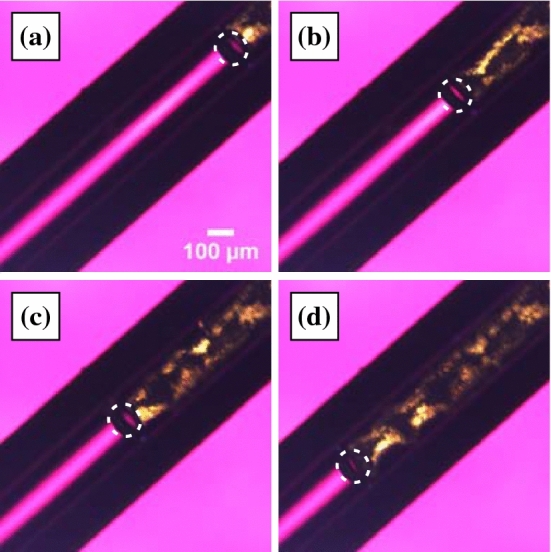
Crystal growth for OTP in a capillary tube with inner diameter 0.13 mm at $T = 283 \,\hbox {K}$. (**a**) $t = 95.7 \,\hbox {s}$, (**b**) 98.7 s, (**c**) 101.7 s, and (**d**) 104.7 s. The dotted lines show the positions of the bubble. Although the crystal is not in contact with the liquid, it was observed that the crystal keeps growing. It is also remarkable that the growth rate is $100.6\, \upmu \hbox {m/s}$, which is close to the filamentous crystal growth rate (see also Fig. 2e).

If this is correct, it should be possible for filamentous crystals to grow even if the liquid and the crystal are not in contact with each other. Figure 5 shows the time evolution of the crystal growth when OTP was placed in a capillary tube with inner diameter 0.13 mm at $T = 283 \,\hbox {K}$. The bubble fills the diameter of the capillary tube and hinders contact between the liquid and the crystal. Although the crystal is not in contact with the liquid, it was observed that the crystal keeps growing. Remarkably, the growth rate is $100.6 \,\upmu \hbox {m/s}$, which is close to the filamentous crystal growth rate (Fig. 2e). Here, we cannot rule out the possibility that the liquid and the crystal are in slight contact. However, the area of contact between the crystal and the liquid is clearly smaller than that in the experiment using a sample confined by flat cover glasses. Since the crystallization rate is unchanged even in the capillary tube, we conclude that the molecules are supplied to the crystal interface through a bubble or layer of gas phase OTP.

In addition, we investigated the crystal growth when the crystal is contacted with an air bubble. We note that it is hard that OTP molecules evaporate to the air bubble. We injected air bubbles into the sample beforehand and observed the crystal growth after a faceted crystal interface makes contact with an air bubble (Movie S4). The size of the air bubble is similar to that of the bubble shown in Fig. 2. In the case of the air bubble, the faceted crystal growth simply continues, with no filaments seen. This means that the evaporation of OTP molecules into the bubbles should be critical for the filamentous crystal growth.

### Trajectory of the bubble motion

We also observed that the bubble motion is not straight but often curved (see Movie S5). The filamentous crystal growth follows the bubble, making bent filaments. It is interesting that an emerged bubble moves in a straight and stable path when the emerged bubble is in contact with two filaments. When one of the two filaments separates from the bubble, the straight motion becomes unstable, and the trajectory starts to curve (see Movie S6).

Here we note that some parameters are complex coupling. As the OTP molecules are evaporated from the liquid and deposited on the crystal on the other side of the bubble, a large temperature gradient is generated inside the bubble due to latent heat. It was reported that the large temperature gradient induces convective Marangoni flow inside the bubble^23,24^. When any inhomogeneities in the temperature profile would misalign the gradient from the growth direction of the filament, the flow inside the bubble should be changed. The flow may affect a deposition rate to the crystal surface. In addition, a contact angle between the bubble and the crystal surface may also affect the bubble motion. When the crystal surface is tilted, the bubble may be deformed or rotated to keep the contact angle. To understand the bubble motion, the microscopic observation and measurement of the local temperature on the bubble surface will be required in future.

### Suppression of the filamentous crystal growth by impurity

**Figure 6:**
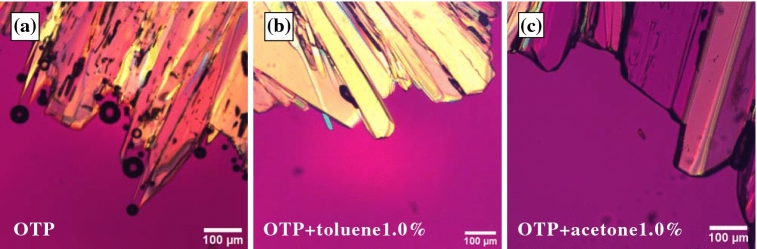
Crystal morphologies in (**a**) pure OTP, (**b**) OTP with 1 wt% toluene, and (**c**) OTP with 1 wt% acetone at 283 K. Filamentous crystal growth with bubbles is observed in pure OTP; when a small amount of toluene or acetone is mixed in, there are no bubbles and only faceted crystal growth is observed.

**Figure 7:**
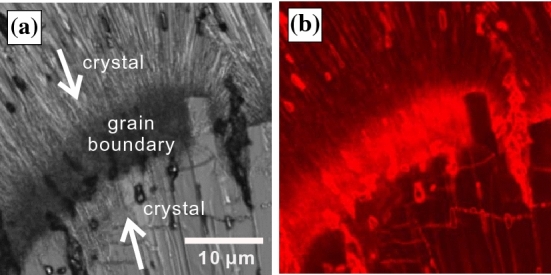
(**a**) Microscopy image and (**b**) fluorescence image of OTP with 1.0 wt% acetone+rhodamine 6G after the crystallization is finished. Those images are taken at the same position. The arrows indicate the directions of the crystal growth. Since the rhodamine 6G is not solved in OTP, the intensity corresponds to the acetone concentration. It is found that the grain boundary is bright, on the other hand, the crystal region is dark.

In the course of the investigation, we also considered the suppressing of the bubble formation by adding a small amount of impurities. We mixed a small amount of organic solvent (toluene or acetone) with OTP as an impurity. Figure 6 shows the crystal growth at 293 K in (a) pure OTP, (b) OTP with 1.0 wt% toluene, and (c) OTP with 1.0 wt% acetone. We confirmed by using DSC measurement that the melting temperature and the glass transition temperature are unchanged when such small amounts of impurities are mixed with OTP. Thus, it is expected that the viscosity is also unchanged. Compared with observations in pure OTP, the number of the bubbles is significantly reduced. Here, we put rhodamine 6G in acetone and then we mixed the acetone with OTP. Fig. 7a shows a microscopy image after the crystallization is finished (Fig. 7a). The arrows in Fig. 7a indicate the directions of the crystal growth and a region filled with small crystallites is a grain boundary. Then Fig. 7b shows a fluorescence image of OTP with 1.0 wt% acetone + rhodamine 6G case at the same position as Fig. 7a. Since the rhodamine 6G is not solved in OTP, the intensity corresponds to the acetone concentration. It is found that the grain boundary is bright, on the other hand, the crystal region is dark. It means that the impurities are accumulated near the crystal growth front. It is expected that the negative pressure near the crystal becomes small since the impurity molecules are accumulated, and then the cavitation is suppressed. Then, faceted crystal growth is only observed when OTP is mixed with impurities.

## Discussion

Here, we discuss physical factors for the filamentous crystal growth rate. The growth rate of the filamentous growth may be determined both by the deposition onto the crystal surface from the bubble and the evaporation from the liquid to the bubble. In this dynamical path, the diffusion in liquid can be ignored and it is consistent that the filamentous crystal growth rate is independent with the viscosity. Here, the amount of the evaporation to the bubble may be proportional to the surface area of the bubble. Thus, a growth rate is larger with a larger bubble in the salol system. However, it is found that the growth rate decreases when the bubble size becomes larger at 278 K in OTP shown as in Fig. 3d. This seems to be because the dynamics of the filamentous growth is not steady in the beginning. The growth rate of the crystal depends on both of the evaporation amount and the deposition one. Both amounts of the evaporation and the deposition depend on the bubble size, the contact area between the bubble and the crystal, and the shape of the bubble. Thus, the velocity may depend on temporal change of those parameters in the beginning. In addition, Fig. 2e shows that the filamentous crystal growth rate suddenly decreases below 278 K, where the emerged bubbles are unstable. The reason for this has been unclear yet, thus it is an important future work.

## Conclusion

To summarize this study, we investigated the mechanism behind filamentous crystal growth in OTP and salol. It was found that bubbles of OTP gas induce filamentous growth. The dynamical pathway is highly out of the ordinary, consisting of evaporation from the liquid to the bubble and subsequent deposition from the bubble to the crystal interface. The mechanism is directly demonstrated in a capillary tube. In addition, by mixing a small amount of an organic solvent such as toluene or acetone, the generation of bubbles is suppressed and filament growth is prevented: this shows that crystal morphology can be selected by mixing (removing) an organic solvent. These results provide new insights into the science of crystal growth. Note that the mechanisms proposed above may be ubiquitous at least in organic liquids. The filamentous crystal growth might be occurred ubiquitously when the density difference between liquid and crystal is large. The results in this paper will greatly contribute to engineering applications such as improving the stability of insulation material, forming crystalline nanowires, and accelerating crystal growth.

## Materials and methods

We used o-terphenyl (OTP) with a purity of 99% purchased from Sigma-Aldrich and phenyl salicylate (salol) with a purity of 98.0% purchased from Wako Pure Chem. Ind.. The melting temperatures $T_m$ of OTP and salol are 331.0 K and 315.0 K, respectively. Before the experiments, OTP and salol are pre-crystallized in small bottles. Impurities was localized at grain boundaries between the crystals. When we slowly heated them above $T_m$, the crystal near the grain boundary was formerly melted. Then we extracted only a crystallizable part to remove impurities. In addition, we annealed the filtered liquid at 400 K for 3 h to remove solved gases in the liquid. We considered that the purity of the materials is much higher than the label displays. A sample was sandwiched between two cover glasses; the sample thickness was set to $25\,\upmu \hbox {m}$ using spacers. The sample was sealed with UV curable glue to prevent air influx and evaporation. The temperature of the sample was controlled using a temperature controller (LINKAM, 10002L). Samples of OTP were quenched to 293 K at 20 K/min, allowing it to completely crystallize. These were heated to $T_m$, where the crystal slowly melted. With a part of the crystal still undissolved, the sample was quenched to a crystallization temperature $T_x$ at 20 K/min. Observation of the samples was initiated at a time $t = 0$, when the temperature reached $T_x$. Note that the undissolved crystalline portions acted as nuclei for growth. The process was observed using a polarizing microscope (Nikon ECLIPSE LV100ND) with a retardation plate; note that the images captured are purple when the sample does not show birefringence. For additional impurities, toluene with a purity of 99.0% purchased from Wako Pure Chem. Ind. and acetone with a purity of 99.0% purchased from Wako Pure Chem. Ind. were mixed with OTP and stirred well. We purchased rhodamine 6G from Wako Pure Chem. Ind. Fluorescence image was taken by a fluorescence microscope (Nikon Eclipse Ni).

## Supplementary Information

Supplementary Video 1.

Supplementary Video 2.

Supplementary Video 3.

Supplementary Video 4.

Supplementary Video 5.

Supplementary Video 6.

## Data Availability

All data generated or analyzed during this study are included in this published article and its supplementary information files.
